# Adiposity and immune-muscle crosstalk in South Asians & Europeans: A cross-sectional study

**DOI:** 10.1038/srep14521

**Published:** 2015-10-12

**Authors:** M.Constantine Samaan, Sonia S. Anand, Arya M. Sharma, Ashley Bonner, Joseph Beyene, Imtiaz Samjoo, Mark A. Tarnopolsky

**Affiliations:** 1 Department of Pediatrics, McMaster University, Hamilton, Ontario, Canada; 2 Division of Pediatric Endocrinology, McMaster Children’s Hospital, Hamilton, Ontario, Canada; 3 Population Genomics Program, Chanchlani Research Centre, McMaster University, Hamilton, ON, Canada; 4 Population Health Research Institute, Hamilton Health Sciences and McMaster University, Hamilton, Ontario, Canada; 5 Department of Medicine, McMaster University, Hamilton, Ontario, Canada; 6 Department of Clinical Epidemiology/Biostatistics, McMaster University, Hamilton, Ontario, Canada; 7 University of Alberta, Edmonton, Alberta, Canada

## Abstract

South Asians (SA) are at higher risk of cardiometabolic disorders than Europeans (EU), yet the potential determinants of this risk are poorly understood. We tested the hypotheses that 1) South Asians (SA) have greater muscle inflammation compared to Europeans (EU) at similar fat mass 2) differential regional adiposity in SA compared to EU is associated with enhanced muscle inflammation in SA. This cross-sectional study was conducted at a tertiary academic center in Hamilton, Ontario, Canada. The study included 29 EU and 26 SA. Quantitative real-time PCR and western blot were used to measure muscle inflammation. Statistical analysis was done using a General Linear Model. Despite having similar macrophage content to EU, SA muscle had lower levels of chemokine CCL2 compared to EU at gene expression (β -1.099, SE β 0.521, p-value 0.04) and protein (0.84 ± 0.69 versus 1.10 ± 0.60, p-value 0.052) levels. SA had more pronounced abdominal and hepatic adiposity, with smaller Intramyocellular lipid particles compared to EU (0.26 ± 0.12 μm^2^ versus 0.15 ± 0.06 μm^2^, p-value 0.02). In conclusion, CCL2 downregulation in SA may be an attempt to protect muscle against macrophage infiltration, and defects in fatty acid partitioning to muscle may lead to the disproportionate adiposity and adverse cardiometabolic profile in SA.

Nearly 2.1 Billion people are overweight or obese worldwide, but obesity affects certain ethnic groups disproportionately^1^,^2^. South Asians (SA), encompassing people originating from India, Pakistan, Sri Lanka and Bangladesh with a significant global diaspora^3^,^4^,^5^, have higher rates of obesity, type 2 diabetes and cardiovascular disease at lower body mass index (BMI) compared to Caucasian Europeans (EU)^6^,^7^. At similar BMI levels, SA have higher visceral adiposity compared to EU^8^. In addition, one study suggested that SA have higher Intramyocellular lipids (IMCL) than EU^9^.

The expansion of different fat depots leads to distinctive consequences on metabolic profiles. Higher visceral adiposity is associated with immune system activation and adipose tissue inflammation^10^,^11^,^12^,^13^,^14^,^15^,^16^,^17^,^18^,^19^, which can be explained by excess fatty acids, cytokines and chemokines in adipose tissue attracting circulating immune cells, including monocytes, neutrophils, and T-Lymphocytes^10^,^11^,^12^,^13^,^14^,^15^,^16^,^17^,^18^,^19^. Monocyte attraction into tissues depends on several chemokines, and one of the most important chemokines involved in this process is Chemokine (C-C motif) Ligand 2^19^. Monocytes will sense local adipose tissue milieu and differentiate to macrophages that secrete inflammation-propagating cytokines^14^,^20^,^21^,^22^. On the other hand, the subcutaneous adipose tissue compartment has been proposed to have favorable metabolic characterisitics, but is SA is characterized by increased adipocyte size and low-grade systemic inflammation with insulin resistance^23^,^24^.

While studies have focused on understanding inflammation in adipose tissue^10^,^11^,^12^,^13^,^14^,^15^,^16^,^17^,^18^, and showed an excess of macrophages in obese adipose tissue in EU, there are no data regarding the effect of systemic (total fat mass) and regional adiposity, including visceral adipose tissue (VAT), intrahepatocellular fat, and intramyocellular lipids (IMCL) on inflammation in skeletal muscle in SA compared to EU^25^,^26^,^27^. As muscle is the supreme metabolic tissue for postprandial glucose uptake^28^,^29^,^30^, understanding the mechanisms of muscle inflammation may help manage its adverse effects on muscle insulin signaling and myocellular fat metabolism, and develop better understanding of the potential contribution of inflammation to insulin resistance and excess cardiometabolic risk in SA compared to EU.

In this study, we tested the association of systemic and regional adiposity with muscle inflammation in SA and EU. We hypothesized that 1) SA have greater muscle inflammation compared to EU at similar total fat mass, and 2) Differential regional adiposity in SA is associated with enhanced muscle inflammation compared to EU.

The primary aim of this study is to determine if SA have more muscle inflammation compared to EU. The secondary aims of this study are to determine if SA have differential adiposity patterns compared to EU, and if these depots are associated with a specific inflammatory profile in muscle that differs between SA and EU.

## Results

### Clinical & biochemical characteristics of participants

Table 1 compares the clinical and biochemical details of SA (n = 26, 7 female) and EU (n = 29, 16 female).

Fitness levels were equal in both groups (VO2 (ml/kg) SA 4.14 ± 1.08 versus EU 4.29 ± 1.17, p-value 0.56). SA had a higher trend for fasting blood glucose (5.00 ± 0.55 mmol/l versus 4.80 ± 0.44 mmol/l, p-value 0.06), HOMA-IR (2.45 ± 1.70 versus 1.70 ± 1.30, p-value 0.07), triglycerides (1.57 ± 1.16 mmol/l versus 1.12 ± 0.60, p-value 0.06) and lower HDL (1.20 ± 0.27 mmol/l versus 1.39 ± 0.37 mmol/l, p-value 0.08) compared to EU.

### Ethnic differences in fat depots

In the overall molSHARE study (n = 108), SA had greater fat mass, intrahepatic and VAT with higher rates of insulin resistance, lower HDL and higher triglycerides compared to EU^8^. In the subgroup of participants included in the current study, SA and EU have similar BMI (26.2 ± 3.5 versus 27.3 ± 5.2, p-value 0.39) and fat mass (23.9 ± 8.1 kg versus 25.7 ± 13.1 kg, p-value 0.53) (Table 1), which allows the comparison of the effects of individual fat depots on muscle inflammation between ethnic groups.

**Figure 1:**
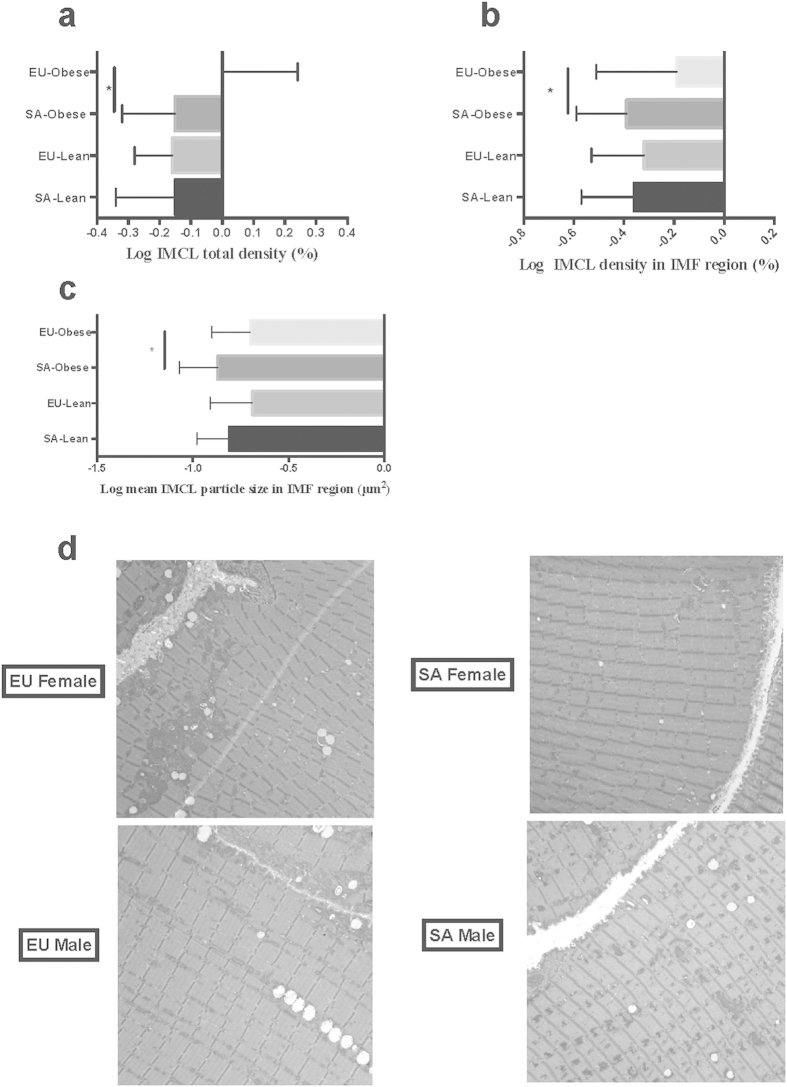
Obese SA have smaller IMCL particles in IMF compared to EU. (**a**) Log IMCL total density (%); (**b**) Log IMCL density in IMF region (%); (**c**) Log mean IMCL particle size in IMF region (μm^2^); (**d**) Representative electron microscopy images for IMCL for SA and EU males and females.

SA participants have larger VAT depot (141.70 ± 15.10 cm^2^ versus 66.00 ± 14.10 cm^2^, p-value 0.001), and increased intrahepatocellular fat (9.90 ± 1.70% versus 2.50 ± 1.90, p-value 0.005) compared to EU (Table 1). In contrast to higher visceral and hepatic adiposity, SA have decreased IMCL density in the IMF region compared to EU (0.41±0.24% versus 0.66±0.50%, p-value 0.04; Fig. 1 a & b); this is due to smaller IMCL particles in the IMF region in the overweight/obese SA compared to overweight/obese EU in both sexes (0.15±0.06 μm^2^ versus 0.26±0.12 μm^2^, p-value 0.02, Fig. 1c & d), with similar number of particles noted in the IMF region. Lean SA and EU had similar IMCL particle size in IMF region (Table 2). IMCL density in subsarcolemmal region correlated positively with HOMA-IR (p-value 0.04).

### Ethnic differences in muscle inflammation

To test the effect of ethnic variations on muscle inflammation, gene and protein expression analyses were undertaken to measure cytokine, chemokine/chemokine receptor, TLR, and immune cell markers including total macrophage content (CD68), inflammatory macrophages (CD11c), resident macrophages (MRC1) and neutrophils (MPO). In the unadjusted analyses, fat mass percentage (%FM) correlated with several inflammatory and immune markers (TNFα, CCL2, CCL3, IL-8, CCR2, TLR4, NLRP3, CD68, and MPO; Table 3). Higher VAT content is associated with reduced resident macrophages (MRC1) in muscle, and higher VAT and intrahepatocellular fat content is associated with higher muscle neutrophil content (MPO) (Table 3). Interestingly, IMCL did not correlate with the inflammatory and immune cell markers tested. From this point onwards, we included only the markers that showed correlation with the %FM in further analyses.

**Figure 2:**
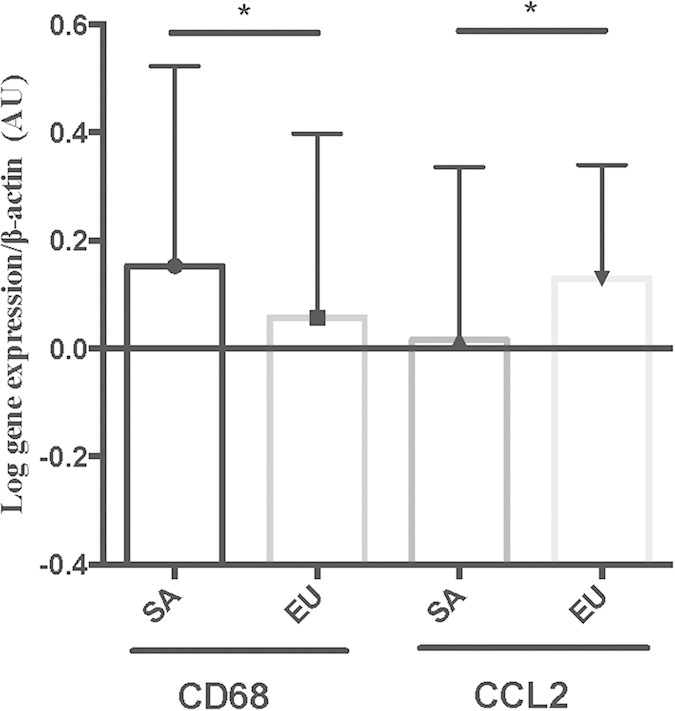
Gene expression analysis of CD68 and CCL2 for SA and EU.

In order to determine the factors influencing the relationship of adiposity (%FM) to muscle inflammation, we included TNFα, CCL2, CCL3, IL-8, CCR2, TLR4, NLRP3, CD68, and MPO in General Linear Model and adjusted for age, sex, BMI, HOMA-IR, fitness and ethnicity. SA had higher CD68 (β 1.411, SE β 0.692, p-value 0.047) and lower CCL2 gene expression (β -1.099, SE β 0.531, p-value 0.040) in muscle when compared to EU (Table 4; Fig. 2). There were no significant associations of VAT, hepatic fat and IMCL with TNFα, CCL2, CCL3, IL-8, CCR2, TLR4, NLRP3, CD68, and MPO (data not shown).

**Figure 3:**
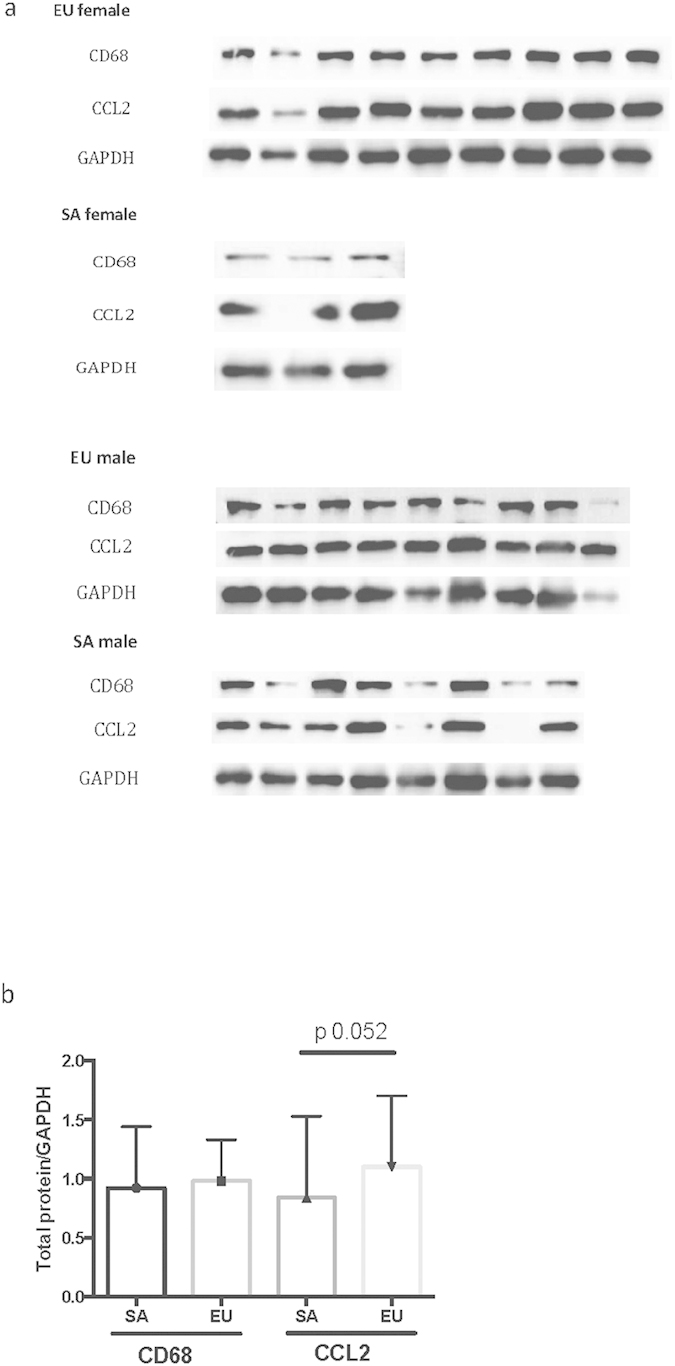
Muscle protein expression of CD68 and CCL2 in SA and EU. (**a**) Western blot data for CD68, CCL2, and GAPDH as control. CD68 produces a band at 110 kDa, CCL2 30 kDa, and GAPDH 37 kDa. (**b**) Quantification of western blot result.

Protein quantification using western blot for CD 68 and CCL2 revealed that CD68 muscle protein content is similar in SA and EU (0.92 ± 0.52 versus 0.98 ± 0.35, p-value 0.63), and there was no correlation between ethnicity and CD68 (p-value 0.37). On the other hand, SA have lower CCL2 muscle protein levels than EU (0.84 ± 0.69 versus 1.10 ± 0.60, p-value 0.052) (Fig. 3a,b). When the whole group is analyzed, neither CD68 nor CCL2 protein correlated with the different fat depots or BMI (data not shown).

## Discussion

In this study of seemingly healthy, non-diabetic subjects with similar age, BMI, and total fat mass, we demonstrate that SA have similar total muscle macrophage content (CD68) and lower monocyte/macrophage chemokine CCL2 compared to EU. SA had smaller IMCL particles in their muscle compared to EU.

In the literature, the evidence describing the relationship of muscle inflammation and adiposity is limited by small sample size, various laboratory techniques used, differences in populations studied, and different statistical methods used for data analysis. Some studies show that muscle macrophages correlate positively with BMI and inversely with insulin sensitivity^25^,^31^,^32^,^33^. In contrast, the association of muscle macrophages with BMI or adiposity has not been replicated in other studies^26^,^27^,^34^. Our results demonstrate that when samples are pooled in the analysis, muscle CD68 content does not correlate with total adiposity, BMI or the different fat depots.

We predicted that the greater VAT and intrahepatic fat depots in SA are associated with a more prominent inflammatory response in muscle compared to EU. Despite stark differences in regional adiposity patterns, no differences are detected in muscle macrophage content, macrophage subtype and other inflammatory markers between SA and EU when VAT, hepatic fat and IMCL are examined for their association with muscle inflammation. This argues for additional indirect mechanisms through which higher regional adiposity associates with muscle inflammation in different ethnic groups, or that inflammation may play a role later once other risk factors e.g. aging, dyslipidemia or dysglycemia are present, as this group is relatively healthy.

It is intriguing that similar CD68 content in SA muscle is witnessed despite the downregulation of one of the main monocyte/macrophage chemokines (CCL2). Polymorphisms in CCL2 gene in SA are associated with enhanced visceral adiposity^35^. There are two possibilities that may explain this paradoxical result of equal macrophage content with lower CCL2 in SA compared to EU. There may be additional chemokines that regulate monocyte/macrophage trafficking into SA muscle, so that even when CCL2 is downregulated these chemokines are upregulated to drive monocyte migration into muscle.

Alternatively, the downregulation of CCL2 in SA is an attempt to protect muscle against further monocyte infiltration. The overexpression of CCL2 in adipose tissue in mice is associated with enhanced monocyte recruitment^36^, and CCL2 knockout mice exhibit reduced adipose inflammation and macrophage infiltration^19^.

We also demonstrate lower IMCL particle size in IMF region in SA compared to EU. This suggests reduced rates of substrate delivery to muscle that may be due to endothelial dysfunction^37^,^38^,^39^,^40^,^41^, and reduced fatty acids available for oxidation. This is consistent with reports of reduced fatty acid oxidation in SA muscle with normal oxidative and lipid metabolism gene expression when compared to EU^37^. Morbidly obese, insulin resistant subjects have reduced fatty acid oxidation, and this is related to insulin resistance^42^. These studies are in contrast to other studies that revealed that SA are capable of increased oxidative capacity in association with insulin resistance^43^. Smaller IMCL particles in SA in our study may explain the differences in ectopic fat deposition between SA and EU. One possibility is that this is an adaptive response by muscle to prevent excess fatty acid supply; the inability to partition excess fatty acids into muscle may then result in larger liver and VAT fat depots, which is associated with adverse cardiometabolic outcomes in SA.

Our results are in contrast to a study that examined IMCL in 20 SA and 20 EU participants by proton magnetic resonance spectroscopy of the soleus muscle, and demonstrated that SA have higher IMCL content than EU^9^. While SA had higher fat mass and lower insulin sensitivity compared to EU, IMCL did not relate to obesity or insulin sensitivity in SA^9^. The difference in results between this study and ours may be explained by the difference in the methods used to quantify IMCL, and the different muscles used in this quantification. IMCL is highest in type I fibers (slow twitch, oxidative), and soleous muscle that was used in the above study has more type 1 fibers than the vastus lateralis muscle used in our study^9^,^44^,^45^,^46^,^47^. In conclusion, the different muscles studied, different methodologies in quantifying IMCL, and potential differences in the population sampled may explain the differences in these results. One of the major strengths of our study is that we evaluated the effect of regional fat depots, measured using state of the art methods, on muscle directly as the total fat mass, BMI and insulin resistance are similar between SA and EU.

Our study has several additional strengths. The relatively large sample size, well-characterized fat depots, and detailed muscle gene expression analyses of inflammatory markers, and protein analysis of CD68 and CCL2 provide a comprehensive evaluation of muscle inflammation. In addition, our statistical analyses were rigorously performed with a priori selected confounding factors to account for differences between the two ethnic groups.

Measuring muscle cytokines and lipid intermediates including diacylglycerol and ceramide in muscle would have been advantageous, because they may be associated with muscle inflammation. However, this requires significant amounts of muscle tissue that is difficult to access due to the invasive nature of the procedure, and obtaining a large biopsy from healthy donors are significant challenges.

In addition, another fat depot that we did not study is the intermuscular adipose tissue. Skeletal muscle has its own complement of fibro/adipogenic progenitors (FAPs). They represent a pool of cells that has been recently defined in the perivascular region in human muscle^48^. These cells participate in muscle homeostatic and injury responses in collaboration with myogenic progenitors and macrophages^49^,^50^. These cells contribute to intermuscular adipose tissue, which expands in lean and overweight men with the metabolic syndrome regardless of ethnicity^51^. The intermuscular adipose tissue also expands in other conditions including obesity, excess fatty acids, hypoxia and inflammation^30^.

In addition, recent evidence suggests that obesity can result in sex-specific muscle fiber organization changes. In obese male mice, the major Type I muscle fiber (slow twitch, oxidative) in soleus muscle rises in proportion to adiposity. There is also transformation of Type I fibers to a hybrid I/IIb type (IIb fast twitch, glycolytic) with male obesity that was not seen in female mice^52^. While vastus lateralis is mainly composed of Type I fibers, the changes in intermuscular fat and how they impact fiber type organization, and the ethnic differences in these responses will require further study.

In summary, this study reveals that SA have similar muscle macrophage content to EU despite downregulation of CCL2, an important monocyte chemokine. In addition, the noted ethnic differences in fat deposition into muscle may explain the differential patterns of fat accumulation in metabolic organs, which increases the risk of cardiometabolic problems known to affect SA at higher rates than EU.

Further investigation into the genetic, epigenetic, environmental and lifestyle factors influencing fat depot development and immune cell traffic into muscle in different ethnic groups is warranted, to enhance our understanding of the effects of ethnicity on skeletal muscle inflammation and fatty acid partitioning into metabolic organs. This may allow the design of targeted therapies to address inflammation and fatty acid deposition in muscle as potential mechanisms associated with adverse cardiometabolic outcomes in SA.

## Methods

This was a cross-sectional study conducted using muscle biopsy samples from the Molecular Study of Health and Risk in Ethnic Groups (Mol-SHARE) cohort, the protocol for which has been previously described^8^. The primary objective of Mol-SHARE study is to determine the differences in adiposity, adipose tissue inflammation and morphology, and muscle lipid and fatty acid oxidation and mitochondrial function between SA and EU^8^.

The full study included 18–50 year old subjects (n = 108) who were recruited via mailed notices for the study, and by advertising in temples, Hamilton hospitals, and McMaster University campus. SA and EU subjects who were age, sex and BMI matched, and grouped across different BMI levels (lean = 18.5–25 kg/m^2^, overweight = 25.1–29.9 kg/m^2^, and obese = 30–45 kg/m^2^. We used a subgroup of the total study population for whom muscle tissue was available (SA n = 26, EU n = 29). The Hamilton Health Sciences Research Ethics Board approved the study, and written informed consent was obtained from all participants. The study was conducted in accordance with appropriate clinical practice guidelines and national legal requirements.

### Fat depot quantifications

The initial body composition measurement and fat mass determination was done using DEXA scans. To quantify abdominal visceral and subcutaneous adipose tissue, a T1-weighted MRI image at the level of mid-L4 (TR 400 ms, TE 13 ms) was acquired. The volume of subcutaneous and visceral fat was determined by manual tracing of the areas of fat. Intrahepatocellular lipids were measured using single voxel MRS (TR 1500 ms, TE 30 ms, 8 averages, 1024 data points over 1000 Hz spectral width, 1–2 cc voxel volume, and acquisition time 18 s, without water suppression).

The IMCL compartment was measured using electron microscopy^53^,^54^. Briefly, freshly biopsied muscle tissue was placed into 2% glutaraldehyde and subjected to transmission electron microscopy for quantification of lipid droplet characteristics. These include the number and density of lipid droplets in subsacrcolemmal (SS) and intermyofibrillar (IMF) regions, mean droplet area, and the percentage of lipids touching mitochondria were measured using image Pro Plus software (Media Cybernetics, Silver Springs, MD). Researchers completing the analyses were blinded to the participants’ ethnicity.

### Fitness testing

Fitness testing was done using an electronically braked cycle ergometer (Excalibur Sport, Lode, Groningen, The Netherlands). Subject breathed at rest through a mouthpiece for 5 minutes before commencing the test while seated. Subjects began the cycle test with stage 1 at 15 watts for 5 min, followed by stage 2 at 50 watts for 10 minutes, with total test duration of 15 minutes. Computerized open-circuit gas collection system (Moxus Modulator VO_2_ system with O_2_ analyzer S-3 A/I and CO_2_ analyzer CD-3A, AEI Technologies, Pittsburgh, PA) was used to collect data (Respiratory Exchange Ratio, heart rate, VO_2_, VCO_2_) at all time points.

### Biochemical Analyses

Participants fasted for 12 hours prior to blood sampling. Enzymatic methods were used to measure total serum cholesterol^55^ and glucose^56^. Serum LDL cholesterol was measured using the Friedewald formula^57^, and a homogenous enzymatic colorimetric assay (ROCHE/Hitachi Modular Package Insert) was used to measure HDL cholesterol. Triglycerides were quantified by the enzymatic colorimetric assay on ROCHE/Hitachi Modular instrument and reagent kit. Insulin was quantified using the Roche Elecsys R 2010 immunoassay analyzer with electrochemiluminescence immunoassay (Roche Diagnostics GmbH, Indianapolis, Indiana, USA). Serum C-Reactive Protein (CRP) was measured using the Roche Hitachi 917 using the Tina-quant R CRP high sensitivity assay.

### Gene expression analysis

A Vastus lateralis muscle biopsy was performed as described previously^8^. Muscle was powdered in liquid nitrogen and added to 1 ml of Trizol. The sample was then homogenized in Trizol and RNA isolation was completed using Qiagen RNAeasy mini kits (Qiagen, Valencia, CA) following manufacturer’s instructions. The quality of the RNA was tested using nanospectrophotometer and all RNA samples had 260/280 ratio of 1.8–2.0. cDNA generation was performed with SuperScript^®^ III reverse transcriptase kit (Invitrogen, Carlsbad, CA) following DNase treatment.

Gene expression analysis was performed for cytokines including Interleukin-6 (IL-6), Tumor Necrosis Factor-alpha (TNFα), Interleukin-10 (IL-10), and chemokines including Chemokine (C-C motif) Ligand 2 (CCL2), Chemokine (C-C motif) Ligand 3 (CCL3), Interluekin-8 (IL-8), and Chemokine (C-C motif) Ligand 20 (CCL20), along with the chemokine receptor for CCL2 called Chemokine (C-C motif) Receptor 2 (CCR2). Innate immune system components were also tested including Toll-Like Receptors 2 & 4 (TLR2 & TLR4).

Innate immune cells including total macrophages (CD68), inflammatory macrophages/dendritic cell (CD11c), resident macrophages (Mannose Receptor, C Type 1, MRC1) and neutrophils (Myeloperoxidase, MPO) were examined. The NACHT, LRR and PYD domains-containing protein 3 (NLRP3) inflammasome and cytokines produced by its activation, including Interluekin-1β (IL-1β) and Interleukin-18 (IL-18) were measured.

Quantitative Real-Time Polymerase Chain Reaction (qRT-PCR) was conducted using TaqMan^®^ Gene Expression Assays (Applied Biosystems; Foster City, CA) using the Rotor-Gene 6000 qRT-PCR machine (Corbett Research; Mortlake, Australia). β-actin was chosen as the reference gene after testing additional reference genes including Ribosomal Protein, Large, P0 (RPLP0) and TATA-box Binding Protein and gene expression was computed using the ΔΔCt method^58^. The chemokine CCL20 was tested but did not result in a PCR product.

### Western blot

The quantification of CD68 and CCL2 protein expression was done using western blot. Briefly, 20 mg of protein were loaded on a 7.5% Tris-Glycine gel using BioRad Mini-PROTEAN Tetra cell electrophoresis chamber. Protein transfer from the gel to nitrocellulose membrane was performed using the BioRad Mini-Transblot Cell running at 100 V for 1 hour at 4 °C. Blots were blocked with 5% Bovine Serum Albumin in TBST buffer with shaking for 1 hour at room temperature. Membranes were incubated with primary CD68 and CCL2 antibodies (Pierce) or GAPDH (Cell Signalling) that were diluted 1:1000 in 5% BSA in TBST overnight at 4 °C. Blots were then washed thrice with TBST, and were then incubated with HRP conjugated secondary antibody (1:1000), with shaking for 1 hour at room temperature and then washed thrice with TBST. Blots were treated with Amersham ECL Western Blotting Detection Reagents for 1 minute and signals detected using Vilber Fusion Imaging machine and software. All bands were quantified using Image J software^59^.

### Statistical analyses

Statistics were performed using SPSS version 20.0 (IBM Corp, Armonk, NY) and Stata/SE 12.1 (Stata Corporation, College Station, TX). Data were examined for normality using the Shapiro-Wilk test, and log transformed if not normally distributed. We imputed missing values of inflammatory markers based on the mean value of the specific inflammatory marker within the corresponding ethnic group. Testing for co-linearity of the cytokine genes and covariates was done using variance inflation factor.

An independent sample t-test was used to examine the differences among clinical variables and metabolic data. A General linear model (GLM) was used to test the association of cytokine gene expression with fat mass percentage (%FM), visceral adipose tissue (VAT), intrahepatocellular fat, and IMCL. The analysis was done initially with no adjustment, and inflammatory and immune markers that correlated with the fat mass with a p-value of 0.1 or less were included in the adjusted analysis, with inclusion of covariates including age, sex, BMI, homeostatic model assessment-insulin resistance (HOMA-IR), fitness levels and ethnicity in the model. Because we had a priori hypothesized the role of the different biomarkers chosen in muscle-immune crosstalk, adjustments for multiple testing are not performed when assessing the significance of these relationships^60^. Quantification of western blots was adjusted for age, sex and ethnicity. Data are reported as mean ± SD unless otherwise stated, and statistical significance was set at p-value of <0.05.

## Additional Information

**How to cite this article**: Samaan, M.C. *et al.* Adiposity and immune-muscle crosstalk in South Asians & Europeans: A cross-sectional study. *Sci. Rep.* **5**, 14521; doi: 10.1038/srep14521 (2015).

## Figures and Tables

**Table 1:** Clinical & biochemical characteristics for participants.

Variable	SA	EU	T-test(p-value)
Total number	26	29	
Age (years)	35.30 ± 11.10	32.20 ± 10.30	0.27
Female (n)	7	16	0.08
Overweight/Obese (n)	15	22	
Systolic BP (mmHg)	112.50 ± 10.20	110.80 ± 9.50	0.49
Diastolic BP (mmHg)	73.70 ± 9.00	73.10 ± 6.50	0.65
Body composition & adiposity			
BMI (kg/m^2^)	26.20 ± 3.50	27.30 ± 5.20	0.39
WHR	0.89 ± 0.09	0.84 ± 0.07	0.016
Fat mass (kg)	23.90 ± 8.10	25.70 ± 13.10	0.53
Fat mass %	32.0 ± 9.20	32.20 ± 13.20	0.95
Lean mass (kg)	50.50 ± 9.50	51.90 ± 10.00	0.58
Visceral adipose tissue area (cm^2^, N = 55)	141.70 ± 15.10	66.00 ± 14.10	0.001
Subcutaneous adipose tissue area (cm^2^, N = 38)	219.10 ± 82.40	244.60 ± 105.00	0.31
Intrahepatocellular fat %	9.90 ± 1.70	2.50 ± 1.90	0.005
Biochemical measurements			
Fasting glucose (mmol/l)	5.00 ± 0.55	4.80 ± 0.44	0.06
Fasting insulin (pmol/l)	8.80 ± 4.10	8.00 ± 5.80	0.26
HOMA-IR	2.45 ± 1.70	1.70 ± 1.30	0.07
Cholesterol (mmol/l)	4.60 ± 0.99	4.80 ± 1.05	0.43
Triglycerides (mmol/l)	1.57 ± 1.16	1.12 ± 0.60	0.06
HDL (mmol/l)	1.20 ± 0.27	1.39 ± 0.37	0.08
LDL (mmol/l)	2.70 ± 0.80	2.80 ± 0.80	0.58
Apo B (mmol/l)	0.91 ± 0.30	0.93 ± 0.29	0.80

SA = South Asian; EU = European; BMI = body mass index; WHR = waist-to-hip ratio; BP = blood pressure; HOMA-IR = Homeostatic model assessment-insulin resistance; HDL = high-density lipoprotein; LDL=low-density lipoprotein; Apo B = Apolipoprotein B; V02 max = maximal oxygen uptake.

**Table 2:** Log IMCL density, particle size and number in lean and obese SA and EU.

Log Variable	Lean								Overweight/obese							
	SA	SD	EU	SD	Mean difference	95%CI		P-value	SA	SD	EU	SD	Mean difference	95%CI		P-value
**IMCL density in SS region (%)**	0.74	0.15	0.62	0.18	0.12	−0.03	0.27	0.11	0.69	0.29	0.82	0.27	−0.13	−0.33	0.07	0.19
**IMCL density in IMF region (%)**	−0.36	0.21	−0.32	0.21	−0.04	−0.22	0.15	0.70	−0.39	0.20	−0.19	0.32	−0.19	−0.37	−0.01	0.04
**IMCL total density (%)**	−0.15	0.19	−0.16	0.12	0.01	−0.13	0.15	0.88	−0.15	0.17	0.001	0.24	−0.15	−0.30	−0.004	0.045
**Mean IMCL particle size in SS region (μm**^**2**^)	−0.63	0.17	−0.54	0.10	−0.09	−0.21	0.04	0.17	−0.65	0.19	−0.67	0.22	0.02	−0.13	0.17	0.79
**Mean IMCL particle size in IMF region (μm**^**2**^)	−0.81	0.17	−0.69	0.22	−0.12	−0.30	0.06	0.18	−0.87	0.20	−0.70	0.20	−0.17	−0.31	−0.03	0.02
**Mean total IMCL particle size (μm**^**2**^)	−0.72	0.14	−0.64	0.18	−0.08	−0.29	0.06	0.27	−0.78	0.18	−0.67	0.19	−0.11	−0.24	0.03	0.12
**IMCL number of particles in SS region (#/μm**^**2**^)	−0.58	0.15	−0.65	0.11	−0.08	−0.22	0.06	0.23	−0.64	0.18	−0.64	0.14	−0.01	−0.12	0.10	0.90
**IMCL number of particles in IMF region (#/μm**^**2**^)	−1.60	0.19	−1.56	0.20	−0.04	−0.21	0.14	0.66	−1.52	0.16	−1.50	0.20	−0.02	−0.15	0.11	0.70
**Mean total IMCL particle (#/μm**^**2**^)	−1.44	0.16	−1.41	0.16	−0.03	−0.18	0.12	0.65	−1.39	0.13	−1.38	0.16	−0.002	−0.10	0.10	0.97

SS = subsacrcolemmal; IMF = intermyofibrillar; IMCL = intramyocellular lipids.

**Table 3:** Unadjusted univariate analysis of muscle gene expression of immune and inflammatory markers correlated with different fat depots.

Marker	%FM			IMCL			VAT			Intrahepatocellular fat		
	β	SE β	P-value	β	SE β	P-value	β	SE β	P-value	β	SE β	P-value
Cytokines												
TNFα	−0.014	0.006	0.02	0.073	0.139	0.60	0.001	0.001	0.92	0.000	0.009	1.00
IL-6	0.000	0.008	0.98	0.117	0.185	0.53	0.001	0.001	0.25	0.017	0.011	0.14
IL-10	0.003	0.012	0.83	0.061	0.256	0.81	0.001	0.001	0.64	0.014	0.016	0.39
Chemokine/receptor												
CCL2	0.017	0.007	0.02	0.209	0.166	0.21	0.000	0.001	0.69	0.010	0.010	0.34
CCL3	−0.019	0.010	0.08	0.349	0.228	0.13	0.001	0.001	0.50	0.006	0.014	0.66
IL-8	0.018	0.010	0.09	0.311	0.225	0.17	0.001	0.001	0.67	0.001	0.014	0.97
CCR2	0.015	0.007	0.04	0.069	0.155	0.66	0.000	0.001	0.76	0.001	0.010	0.95
TLRs												
TLR2	0.004	0.008	0.57	0.213	0.171	0.22	0.000	0.001	0.89	0.001	0.011	0.96
TLR4	−0.017	0.009	0.07	0.099	0.205	0.63	0.000	0.001	0.83	0.004	0.013	0.73
Inflammasome												
IL-1β	−0.007	0.010	0.48	0.170	0.212	0.43	0.001	0.001	0.25	0.015	0.013	0.24
IL-18	−0.009	0.012	0.45	0.076	0.264	0.78	0.002	0.001	0.13	0.004	0.016	0.82
NLRP3	−0.023	0.010	0.02	0.279	0.225	0.22	0.001	0.001	0.34	0.018	0.014	0.19
Immune cells												
CD68	−0.020	0.010	0.05	0.217	0.224	0.34	0.001	0.001	0.55	0.009	0.014	0.50
MRC1	−0.010	0.008	0.23	0.223	0.185	0.23	0.003	0.001	0.01	0.007	0.012	0.55
CD11c	−0.011	0.007	0.13	0.258	0.159	0.11	0.000	0.001	0.65	0.007	0.010	0.52
MPO	0.065	0.017	0.0003	0.017	0.424	0.97	0.004	0.002	0.08	0.044	0.025	0.09

**Table 4:** South Asians have higher muscle CD68 and lower CCL2 gene expression compared to Europeans.

Marker	β	SE β	P-value
CD68	1.411	0.692	0.047
CCR2	0.041	0.533	0.939
MPO	−0.584	1.031	0.574
TNFα	−0.284	0.408	0.491
CCL2	−1.099	0.521	0.040
CCL3	−0.037	0.735	0.960
IL-8	−0.651	0.795	0.417
TLR4	0.585	0.700	0.408
NLRP3	0.105	0.768	0.892

General linear model addressing the relationship between inflammatory markers and %FM in South Asians compared to Europeans adjusted for age, sex, BMI, HOMA-IR, fitness and ethnicity.

## References

[b1] Campos P., Saguy A., Ernsberger P., Oliver E. & Gaesser G. The epidemiology of overweight and obesity: public health crisis or moral panic? International journal of epidemiology 35, 55–60, 10.1093/ije/dyi254 (2006). 16339599

[b2] Ng M. *et al.* Global, regional, and national prevalence of overweight and obesity in children and adults during 1980-2013: a systematic analysis for the Global Burden of Disease Study 2013. The Lancet 384, 766–781, 10.1016/S0140-6736(14)60460-8 (2014). PMC4624264 24880830

[b3] de Munter J. S., Agyemang C., van Valkengoed I. G., Bhopal R. & Stronks K. Sex difference in blood pressure among South Asian diaspora in Europe and North America and the role of BMI: a meta-analysis. J Hum Hypertens 25, 407–417, 10.1038/jhh.2010.77 (2011). 20686502

[b4] Madrigal L. *et al.* Obesity, hypertension, and migration: a meta-analysis of populations of the South Asian diaspora. Human biology 83, 71–86, 10.3378/027.083.0105 (2011). 21453005

[b5] Patel K. C. & Bhopal R. Diabetes epidemic in the South Asian Diaspora: action before desperation. J R Soc Med 100, 115–116, 10.1258/jrsm.100.3.115 (2007). 17339300 PMC1809161

[b6] Anand S. S. *et al.* Differences in risk factors, atherosclerosis, and cardiovascular disease between ethnic groups in Canada: the Study of Health Assessment and Risk in Ethnic groups (SHARE). The Lancet 356, 279–284, 10.1016/S0140-6736(00)02502-2 (2000). 11071182

[b7] Yusuf S., Reddy S., Ounpuu S. & Anand S. Global burden of cardiovascular diseases: Part II: variations in cardiovascular disease by specific ethnic groups and geographic regions and prevention strategies. Circulation 104, 2855–2864, 10.1161/hc4701.099488 (2001). 11733407

[b8] Anand S. S. *et al.* Adipocyte Hypertrophy, Fatty Liver and Metabolic Risk Factors in South Asians: The Molecular Study of Health and Risk in Ethnic Groups (mol-SHARE). PLoS One 6, e22112, 10.1371/journal.pone.0022112 (2011). 21829446 PMC3145635

[b9] Forouhi N. G. *et al.* Relation of triglyceride stores in skeletal muscle cells to central obesity and insulin sensitivity in European and South Asian men. Diabetologia 42, 932–935, 10.1007/s001250051250 (1999). 10491752

[b10] Samaan M. C. The macrophage at the intersection of immunity and metabolism in obesity. Diabetology & Metabolic Syndrome 3, 29, 10.1186/1758-5996-3-29 (2011). 22035457 PMC3223491

[b11] Kim C. S. *et al.* Circulating levels of MCP-1 and IL-8 are elevated in human obese subjects and associated with obesity-related parameters. Int J Obes (Lond) 30, 1347–1355, 10.1038/sj.ijo.0803259 (2006). 16534530

[b12] Weisberg S. P. *et al.* Obesity is associated with macrophage accumulation in adipose tissue. J Clin Invest 112, 1796–1808, 10.1172/JCI19246112/12/1796 (2003). 14679176 PMC296995

[b13] Shoelson S. E., Herrero L. & Naaz A. Obesity, inflammation, and insulin resistance. Gastroenterology 132, 2169–2180, S0016-5085(07)00585-910.1053/j.gastro.2007.03.059 (2007). 17498510 10.1053/j.gastro.2007.03.059

[b14] Lumeng C. N., Deyoung S. M., Bodzin J. L. & Saltiel A. R. Increased inflammatory properties of adipose tissue macrophages recruited during diet-induced obesity. Diabetes 56, 16–23, 56/1/1610.2337/db06-1076 (2007). 17192460 10.2337/db06-1076

[b15] Nguyen M. T. *et al.* A subpopulation of macrophages infiltrates hypertrophic adipose tissue and is activated by free fatty acids via Toll-like receptors 2 and 4 and JNK-dependent pathways. J Biol Chem 282, 35279–35292, 10.1074/jbc.M706762200 (2007). 17916553

[b16] Xu H. *et al.* Chronic inflammation in fat plays a crucial role in the development of obesity-related insulin resistance. J Clin Invest 112, 1821–1830, 10.1172/JCI19451112/12/1821 (2003). 14679177 PMC296998

[b17] Elgazar-Carmon V., Rudich A., Hadad N. & Levy R. Neutrophils transiently infiltrate intra-abdominal fat early in the course of high-fat feeding. J Lipid Res 49, 1894–1903, M800132-JLR20010.1194/jlr.M800132-JLR200 (2008). 18503031 10.1194/jlr.M800132-JLR200

[b18] Kintscher U. *et al.* T-lymphocyte infiltration in visceral adipose tissue: a primary event in adipose tissue inflammation and the development of obesity-mediated insulin resistance. Arterioscler Thromb Vasc Biol 28, 1304–1310, ATVBAHA.108.165100 10.1161/ATVBAHA.108.165100 (2008). 18420999

[b19] Kanda H. *et al.* MCP-1 contributes to macrophage infiltration into adipose tissue, insulin resistance, and hepatic steatosis in obesity. J Clin Invest 116, 1494–1505, 10.1172/JCI26498 (2006). 16691291 PMC1459069

[b20] Lumeng C. N., DelProposto J. B., Westcott D. J. & Saltiel A. R. Phenotypic switching of adipose tissue macrophages with obesity is generated by spatiotemporal differences in macrophage subtypes. Diabetes 57, 3239–3246, db08-087210.2337/db08-0872 (2008). 18829989 10.2337/db08-0872 PMC2584129

[b21] Nayak B. S. & Roberts L. Relationship between inflammatory markers, metabolic and anthropometric variables in the Caribbean type 2 diabetic patients with and without microvascular complications. J Inflamm (Lond) 3, 17, 10.1186/1476-9255-3-17 (2006). 17187674 PMC1764741

[b22] Nayak B. S. *et al.* Association of low serum creatinine, abnormal lipid profile, gender, age and ethnicity with type 2 diabetes mellitus in Trinidad and Tobago. Diabetes Res Clin Pract 91, 342–347, 10.1016/j.diabres.2010.12.017 (2011). 21208679

[b23] Chandalia M. *et al.* Insulin resistance and body fat distribution in South Asian men compared to Caucasian men. PLoS One 2, e812, 10.1371/journal.pone.0000812 (2007). 17726542 PMC1950568

[b24] Garg A. Regional adiposity and insulin resistance. J Clin Endocrinol Metab 89, 4206–4210, 10.1210/jc.2004-0631 (2004). 15356007

[b25] Varma V. *et al.* Muscle inflammatory response and insulin resistance: synergistic interaction between macrophages and fatty acids leads to impaired insulin action. Am J Physiol Endocrinol Metab 296, E1300–1310, 90885.200810.1152/ajpendo.90885.2008 (2009). 19336660 10.1152/ajpendo.90885.2008 PMC2692398

[b26] Bruun J. M., Helge J. W., Richelsen B. & Stallknecht B. Diet and exercise reduce low-grade inflammation and macrophage infiltration in adipose tissue but not in skeletal muscle in severely obese subjects. Am J Physiol Endocrinol Metab 290, E961–967, 00506.2005 10.1152/ajpendo.00506.2005 (2006). 16352667

[b27] Di Gregorio G. B. *et al.* Expression of CD68 and macrophage chemoattractant protein-1 genes in human adipose and muscle tissues: association with cytokine expression, insulin resistance, and reduction by pioglitazone. Diabetes 54, 2305–2313, 54/8/2305 (2005). 16046295 10.2337/diabetes.54.8.2305

[b28] Petersen K. F. & Shulman G. I. Pathogenesis of skeletal muscle insulin resistance in type 2 diabetes mellitus. Am J Cardiol 90, 11G–18G (2002). 10.1016/s0002-9149(02)02554-7 12231074

[b29] Godoy-Matos A. F. *et al.* Rosiglitazone decreases intra- to extramyocellular fat ratio in obese non-diabetic adults with metabolic syndrome. Diabetic medicine : a journal of the British Diabetic Association 27, 23–29, 10.1111/j.1464-5491.2009.02868.x (2010). 20121885

[b30] Vettor R. *et al.* The origin of intermuscular adipose tissue and its pathophysiological implications. Am J Physiol Endocrinol Metab 297, E987–998, 10.1152/ajpendo.00229.2009 (2009). 19738037

[b31] Kim T. H. *et al.* IL-6 induction of TLR-4 gene expression via STAT3 has an effect on insulin resistance in human skeletal muscle. Acta diabetologica 50, 189–200, 10.1007/s00592-011-0259-z (2013). 21293887

[b32] Fink L. N. *et al.* Pro-inflammatory macrophages increase in skeletal muscle of high fat-fed mice and correlate with metabolic risk markers in humans. Obesity 22, 747–757, 10.1002/oby.20615 (2014). 24030890

[b33] Fink L. N. *et al.* Expression of anti-inflammatory macrophage genes within skeletal muscle correlates with insulin sensitivity in human obesity and type 2 diabetes. Diabetologia 56, 1623–1628, 10.1007/s00125-013-2897-x (2013). 23595247

[b34] Tam C. S. *et al.* Low macrophage accumulation in skeletal muscle of obese type 2 diabetics and elderly subjects. Obesity (Silver Spring) 20, 1530–1533, 10.1038/oby.2012.24 (2012). 22314623 PMC3561725

[b35] Kaur S. *et al.* Association of monocyte chemoattractant protein-1-2518 polymorphism with metabolic syndrome in a South Indian cohort. Metabolic Syndrome and Related Disorders 7, 193–198, 10.1089/met.2008.0064 (2009). 19450143

[b36] Kamei N. *et al.* Overexpression of monocyte chemoattractant protein-1 in adipose tissues causes macrophage recruitment and insulin resistance. J Biol Chem 281, 26602–26614, M60128420010.1074/jbc.M601284200 (2006). 16809344 10.1074/jbc.M601284200

[b37] Hall L. M. L. *et al.* Fat Oxidation, Fitness and Skeletal Muscle Expression of Oxidative/Lipid Metabolism Genes in South Asians: Implications for Insulin Resistance? PLoS One 5, e14197, 10.1371/journal.pone.0014197 (2010). 21152018 PMC2995737

[b38] Saltin B., Kiens B., Savard G. & Pedersen P. K. Role of hemoglobin and capillarization for oxygen delivery and extraction in muscular exercise. Acta physiologica Scandinavica. Supplementum 556, 21–32 (1986). 3471054

[b39] Hepple R. T., Mackinnon S. L., Goodman J. M., Thomas S. G. & Plyley M. J. Resistance and aerobic training in older men: effects on VO2peak and the capillary supply to skeletal muscle. J Appl Physiol (1985) 82, 1305–1310 (1997). 9104869 10.1152/jappl.1997.82.4.1305

[b40] Lillioja S. *et al.* Skeletal muscle capillary density and fiber type are possible determinants of *in vivo* insulin resistance in man. J Clin Invest 80, 415–424, 10.1172/JCI113088 (1987). 3301899 PMC442253

[b41] Lithell H. *et al.* Body weight, skeletal muscle morphology, and enzyme activities in relation to fasting serum insulin concentration and glucose tolerance in 48-year-old men. Diabetes 30, 19–25 (1981). 7014301 10.2337/diab.30.1.19

[b42] Coen P. M. *et al.* Reduced skeletal muscle oxidative capacity and elevated ceramide but not diacylglycerol content in severe obesity. Obesity (Silver Spring) 21, 2362–2371, 10.1002/oby.20381 (2013). 23512750 PMC4136513

[b43] Nair K. S. *et al.* Asian Indians have enhanced skeletal muscle mitochondrial capacity to produce ATP in association with severe insulin resistance. Diabetes 57, 1166–1175, 10.2337/db07-1556 (2008). 18285554 PMC7812549

[b44] Jansson E., Esbjornsson M., Holm I. & Jacobs I. Increase in the proportion of fast-twitch muscle fibres by sprint training in males. Acta Physiol Scand 140, 359–363, 10.1111/j.1748-1716.1990.tb09010.x (1990). 2150579

[b45] Esbjornsson M. *et al.* Muscle fibre types and enzyme activities after training with local leg ischaemia in man. Acta Physiol Scand 148, 233–241, 10.1111/j.1748-1716.1993.tb09554.x (1993). 8213179

[b46] Simoneau J. A. & Bouchard C. Human variation in skeletal muscle fiber-type proportion and enzyme activities. Am J Physiol 257, E567–572 (1989). 2529775 10.1152/ajpendo.1989.257.4.E567

[b47] Hwang J. H., Pan J. W., Heydari S., Hetherington H. P. & Stein D. T. Regional differences in intramyocellular lipids in humans observed by *in vivo* 1H-MR spectroscopic imaging. J Appl Physiol (1985) 90, 1267–1274 (2001). 11247923 10.1152/jappl.2001.90.4.1267

[b48] Uezumi A. *et al.* Identification and characterization of PDGFR[alpha]+ mesenchymal progenitors in human skeletal muscle. Cell death & disease 5, e1186, 10.1038/cddis.2014.161 (2014). 24743741 PMC4001314

[b49] Joe A. W. *et al.* Muscle injury activates resident fibro/adipogenic progenitors that facilitate myogenesis. Nature cell biology 12, 153–163, 10.1038/ncb2015 (2010). 20081841 PMC4580288

[b50] Lemos D. R. *et al.* Nilotinib reduces muscle fibrosis in chronic muscle injury by promoting TNF-mediated apoptosis of fibro/adipogenic progenitors. Nat Med, 10.1038/nm.3869 (2015). 26053624

[b51] Goodpaster B. H. *et al.* Obesity, regional body fat distribution, and the metabolic syndrome in older men and women. Arch Intern Med 165, 777–783, 10.1001/archinte.165.7.777 (2005). 15824297

[b52] Denies M. S. *et al.* Diet-induced obesity alters skeletal muscle fiber types of male but not female mice. Physiological reports 2, e00204, 10.1002/phy2.204 (2014). 24744883 PMC3967687

[b53] Devries M. C., Lowther S. A., Glover A. W., Hamadeh M. J. & Tarnopolsky M. A. IMCL area density, but not IMCL utilization, is higher in women during moderate-intensity endurance exercise, compared with men. American Journal of Physiology—Regulatory, Integrative and Comparative Physiology 293, R2336–R2342, 10.1152/ajpregu.00510.2007 (2007). 17913867

[b54] Samjoo I. A. *et al.* Markers of skeletal muscle mitochondrial function and lipid accumulation are moderately associated with the homeostasis model assessment index of insulin resistance in obese men. PLoS One 8, e66322, 10.1371/journal.pone.0066322 (2013). 23776659 PMC3680409

[b55] Allain C. C., Poon L. S., Chan C. S., Richmond W. & Fu P. C. Enzymatic determination of total serum cholesterol. Clin Chem 20, 470–475 (1974). 4818200

[b56] Neeley W. E. Simple automated determination of serum or plasma glucose by a hexokinase-glucose-6 -phosphate dehydrogenase method. Clin Chem 18, 509–515 (1972). 5026763

[b57] Friedewald W. T., Levy R. I. & Fredrickson D. S. Estimation of the concentration of low-density lipoprotein cholesterol in plasma, without use of the preparative ultracentrifuge. Clin Chem 18, 499–502 (1972). 4337382

[b58] Livak K. J. & Schmittgen T. D. Analysis of relative gene expression data using real-time quantitative PCR and the 2(-Delta Delta C(T)) Method. Methods 25, 402–408, 10.1006/meth.2001.1262 (2001). 11846609

[b59] Schneider C. A., Rasband W. S. & Eliceiri K. W. NIH Image to ImageJ: 25 years of image analysis. Nat Meth 9, 671–675, 10.1038/nmeth.2089 (2012). PMC5554542 22930834

[b60] Feise R. J. Do multiple outcome measures require p-value adjustment? BMC Med Res Methodol 2, 8, 10.1186/1471-2288-2-8 (2002). 12069695 PMC117123

